# Factors affecting inter-individual variability in endoxifen concentrations in patients with breast cancer: results from the prospective TOTAM trial

**DOI:** 10.1007/s10549-022-06643-y

**Published:** 2022-07-16

**Authors:** C. Louwrens Braal, Justin D. Westenberg, Sanne M. Buijs, Steven Abrams, Tessa A. M. Mulder, Ron H. N. van Schaik, Stijn L. W. Koolen, Agnes Jager, Ron H. J. Mathijssen

**Affiliations:** 1 Department of Medical Oncology, Erasmus MC Cancer Institute, Dr. Molewaterplein 40, PO box 2040, 3015 CN Rotterdam, The Netherlands; 2 Data Science Institute, Interuniversity Institute for Biostatistics and Statistical Bioinformatics, UHasselt, Hasselt, Belgium; 3 Global Health Institute, Family Medicine and Population Health, University of Antwerp, Antwerp, Belgium; 4 Department of Clinical Chemistry, Erasmus MC University Center, Rotterdam, The Netherlands; 5 Department of Hospital Pharmacy, Erasmus MC University Center, Rotterdam, The Netherlands

**Keywords:** Tamoxifen, Endoxifen, Early breast cancer, Predictive modeling, Therapeutic drug monitoring

## Abstract

**Introduction:**

Endoxifen—the principal metabolite of tamoxifen—is subject to a high inter-individual variability in serum concentration. Numerous attempts have been made to explain this, but thus far only with limited success. By applying predictive modeling, we aimed to identify factors that determine the inter-individual variability. Our purpose was to develop a prediction model for endoxifen concentrations, as a strategy to individualize tamoxifen treatment by model-informed dosing in order to prevent subtherapeutic exposure (endoxifen < 16 nmol/L) and thus potential failure of therapy.

**Methods:**

Tamoxifen pharmacokinetics with demographic and pharmacogenetic data of 303 participants of the prospective TOTAM study were used. The inter-individual variability in endoxifen was analyzed according to multiple regression techniques in combination with multiple imputations to adjust for missing data and bootstrapping to adjust for the over-optimism of parameter estimates used for internal model validation.

**Results:**

Key predictors of endoxifen concentration were *CYP2*D6 genotype, age and weight, explaining altogether an average-based optimism corrected 57% (95% CI 0.49–0.64) of the inter-individual variability. *CYP2D6* genotype explained 54% of the variability. The remaining 3% could be explained by age and weight. Predictors of risk for subtherapeutic endoxifen (< 16 nmol/L) were *CYP2D6* genotype and age. The model showed an optimism-corrected discrimination of 90% (95% CI 0.86–0.95) and sensitivity and specificity of 66% and 98%, respectively. Consecutively, there is a high probability of misclassifying patients with subtherapeutic endoxifen concentrations based on the prediction rule.

**Conclusion:**

The inter-individual variability of endoxifen concentration could largely be explained by *CYP2D6* genotype and for a small proportion by age and weight. The model showed a sensitivity and specificity of 66 and 98%, respectively, indicating a high probability of (misclassification) error for the patients with subtherapeutic endoxifen concentrations (< 16 nmol/L). The remaining unexplained inter-individual variability is still high and therefore model-informed tamoxifen dosing should be accompanied by therapeutic drug monitoring.

**Supplementary Information:**

The online version contains supplementary material available at 10.1007/s10549-022-06643-y.

## Introduction

Globally, breast cancer is the most frequent cancer among women and also the most frequent malignancy overall impacting 2.26 million cases annually [1].In approximately 70% of primary breast cancer, the tumor is estrogen- receptor positive (ER+) and is dependent on estrogen for its proliferation [2]. Tamoxifen is a widely used selective estrogen receptor modulator (SERM) against ER+ breast cancer in the adjuvant setting [3]. Tamoxifen substantially reduces the risk of recurrence, breast cancer mortality, and also overall mortality [4–7].

**Fig. 1:**
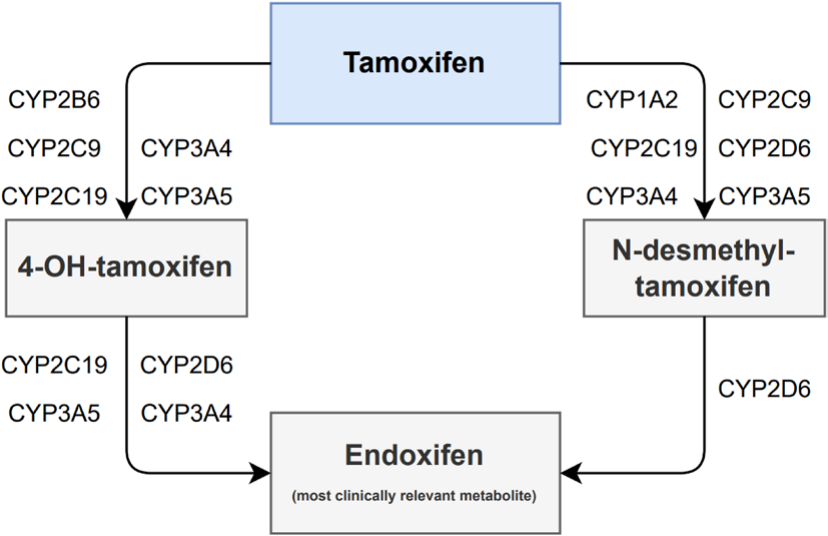
Simplistic representation of biotransformation of tamoxifen into its most clinically relevant metabolite endoxifen

Tamoxifen is a prodrug and is mainly metabolized by the cytochrome P450 (CYP) enzymes 2D6 and 3A4 into its most clinically relevant metabolite endoxifen (Fig. 1) [8]. Endoxifen is characterized by a high inter-individual variability partly due to its complex metabolism [9–11]. To ensure optimal treatment efficacy, a therapeutic endoxifen threshold of 14–16 nmol/L (5.22–5.97 ng/mL) is proposed [9, 12]. However, one out of five patients do not reach therapeutic endoxifen concentrations and thus may fail to retain optimal treatment efficacy [9, 12, 13].

Genetic polymorphisms of the cytochrome P450 (CYP) system—and especially the polymorphic enzyme CYP2D6—are prominently involved in tamoxifen metabolism and consecutively in its high variability in pharmacokinetics [9, 12, 14, 15]. Therefore, numerous studies and guidelines have focused on *CYP2D6* genotype as a predictive marker for individualization of tamoxifen therapy. However, based on the current literature *CYP2D6* genotype accounts for approximately 30–40% of the total variability of endoxifen concentration which calls for further individualization strategies [15–17].

Consequently, several studies have investigated other factors contributing to the inter-individual variability. An important factor explaining the inter-individual variability in endoxifen plasma concentration is adherence to tamoxifen treatment. One-year adherence based on biochemical definition (tamoxifen < 100 nmol/L) and self-reported adherence showed non-adherence in 16% and 12% of the population, respectively [18, 19]. However, real-life estimates range from 15 to 72% non-adherence over 5 years of tamoxifen therapy; with a tamoxifen adherence cut-off in the range of 100–160 nmol/L [20–22].

In the past, age, weight, and body mass index (BMI), amongst others, have been identified as predictors, however, only marginal effects regarding the inter-individual variability in endoxifen concentrations were found. Concurrent medication inhibiting or inducing CYP2D6 enzymatic activity are known to affect endoxifen concentrations and therefore are of clinical value. Nonetheless, with this knowledge, the one-dose-fits-all strategy is still current practice [12, 15, 19, 22–26].

A strategy for individualizing tamoxifen treatment involves the quantification of predictors for endoxifen steady-state concentrations [15–17]. Therefore, the aim of this study was: (i) to quantify the inter-individual variability in endoxifen concentrations in breast cancer patients treated with tamoxifen; (ii) to identify possible predictors of the inter-individual variability in endoxifen concentration; and (iii) to develop a predictive model for endoxifen concentration as a strategy to individualize tamoxifen treatment by model-informed precision dosing.

## Methods

### Study design and population

Participants included in this analysis are selected from the TOTAM study. The TOTAM study is an open-label, single arm, monocentric clinical trial performed at the Erasmus MC – Cancer Institute in Rotterdam, the Netherlands. The primary aim of the TOTAM study is to prove that therapeutic drug monitoring (TDM) of endoxifen for tamoxifen precision dosing is feasible in patients with hormone-sensitive breast cancer in clinical practice. A secondary aim is to develop a model to predict the endoxifen plasma concentration in tamoxifen users. The study protocol is registered in the Netherlands Trial Registry (www.trialregister.nl; NL6918). The TOTAM study prospectively enrolled 314 participants between January 2018 and November 2020. Eligible participants for analysis were ER+ breast cancer patients who initiated a tamoxifen treatment dose of 20 mg administered once daily and reached steady-state concentration (at least 2.5 months of treatment before the first endoxifen measurement). Participants with tamoxifen plasma concentrations < 100 nmol/L were considered non-eligible for analysis due to non-adherence. All participants provided a written informed consent before enrolment.

### Data collection

Data from the first outpatient hospital visit were used, including participants’ demographics, medical history, serum biochemistry, pharmacokinetic sample, and pharmacogenetics. Concurrent use of co-medication or supplements known to strongly inhibit or induce CYP2D6 and CYP3A4 were categorized in weak, moderate and strong inhibitors/inducers, respectively. Tamoxifen-related adverse events were monitored and graded via the Common Terminology Criteria for Adverse Events (CTCAE) version 5 [27]. Adherence to tamoxifen treatment was quantified by the validated 8-item Morisky Medication Adherence Scale; a widely used self-report questionnaire resulting in a high (score 8), medium (score 6–7) or low (score < 5) adherence rate [28].

#### Pharmacokinetics of tamoxifen and endoxifen

Pharmacokinetic blood samples were drawn at steady state to quantify tamoxifen- and endoxifen plasma concentration. Blood samples for the quantification of tamoxifen and endoxifen were all analyzed at the laboratory of Translational Pharmacology, Erasmus MC Cancer Institute, Rotterdam, The Netherlands, using a validated liquid chromatography tandem mass spectrometry method [29].

#### Pharmacogenetics of *CYP2D6* and CYP3A4*22

Participants were *CYP2D6* and CYP3A4*22 genotyped using the Infiniti Biofilm Microarray (Autogenomics Carlsbad, USA) and the Quantstudio test (ThermoFisher Scientific Waltham, USA). CYP2D6 phenotype was assayed in the laboratory on the genetic variants *2 to *10, *12, *14, *17, *29, and *41; thereafter, patients were classified into four phenotypes based on enzyme function. Consecutively, the CYP2D6 activity score (AS) was calculated according to their allele combination and according to the sum of the AS, participants were assigned to four phenotypes: poor metabolizer (PM; AS = 0), intermediate metabolizer (IM; AS ≥ 0.5 to ≤ 1.0), normal metabolizer (NM; AS ≥ 1.5 to ≤ 2.5), and ultra-rapid metabolizer (UM; AS ≥ 3.0). Additionally, all participants were assigned to a phenotype based on their allele combination, including PM/PM, IM/PM, IM/IM, NM/PM, NM/IM, and NM/NM. All participants were genotyped for CYP3A4*22 for wild type (CC), heterozygous (CT), and homozygous (TT). Activity score calculations and phenotyping were in agreement with the Clinical Pharmacogenetic Implementation Consortium guidelines [30].

### Statistical analysis

To summarize baseline characteristics of the study participants, descriptive statistics were computed. Differences between groups were calculated by means of appropriate parametric or non-parametric tests. All tests for differences were two-sided and based on a significance level of 5%. Multiple linear regression was used to model the association between predictors and endoxifen concentration. A logarithmic transformation was applied to endoxifen concentrations to adjust for non-normality. The following variables were included in the regression analysis: age (years), weight (kg), BMI (kg/m^2^), *CYP2D6*, CYP3A4*22, co-medication (CYP2D6 and CYP3A4/5 inhibitors/inducers), intake-with-food, time-of-intake and adherence to tamoxifen.

In order to account for missing data, Multiple Imputation by Chained Equations (MICE) to create multiple complete datasets was performed. A standard Multiple Imputation (MI) scheme was considered and consisted of (i) imputation of all the missing data *m* times, (ii) analysis of *m* imputed datasets, and (iii) pooling of the parameters across *m* analyses according to Rubin’s Rules [31, 32]. Imputation of missing data was according to a full conditional specification approach as variables with missing data were measured at different scales. To incorporate model selection in the MI scheme, a stepwise selection procedure was implemented.

The first step incorporated performing model selection on each imputed dataset separately by means of multiple linear regression with backwards selection based on two stopping rules—namely a significance level of *p* < 0.10 and the Akaike Information Criteria (AIC) [32–34]. Consecutively, a majority rule is applied, i.e., predictors are included in the final model when they were selected in at least 50% of the intermediate models across all MI datasets. Wald-based statistics were computed for variables with selection probabilities between 0.4 and 0.6 to compare two nested models for improvement and thereby yield the exclusion of non-predictive variables [32, 35]. A non-parametric bootstrap procedure was nested within MI approach to correct for model optimism due to overfitting. After creating *m* completed datasets 5000 bootstrap samples were drawn, for each dataset, before the results were pooled [32, 36, 37]. Consecutively, pooled parameter estimates and average-based optimism-adjusted *R*^2^ values were calculated including average bootstrap-based 95% confidence intervals (CIs). The mice package (R Statistics) was used to estimate model parameters. More information regarding model selection procedure can be found in the supplementary material (sects. 5.1 and 5.2).

As a sensitivity analysis, a complete case analysis (CCA) was performed by means of multiple linear regression with backwards selection based on two stopping rules—namely a significance level of *p* < 0.10 and AIC [33, 34]. In order to formally test for improvement in nested models, a Wald test was applied [34]. Again, to adjust for model optimism, a non-parametric bootstrap including 5000 bootstrap samples was performed to estimate parameters and optimism-adjusted *R*^2^ with accompanying 95% CIs. Goodness-of-fit plots were computed to assess model performance and to check model assumptions such as normality, linearity, no or minimal multicollinearity, and heteroscedasticity of residuals.

Finally, key predictors for subtherapeutic risk of endoxifen concentration (< 16 nmol/L) were identified by means of multiple logistic regression. The likelihood ratio test was used to identify the best fitting model, thereby excluding non-predictive variables. The area under the receiver operator curve (AUC) was computed and a bootstrap approach was considered to estimate optimism-adjusted predictive performance measures. All statistical analyses were carried out using the statistical software package R (R statistics. Foundation of Statistical Computing©, version 3.5.4.).

## Results

### Patients and data

Between January 2018 and November 2020, 314 early breast cancer patients were enrolled in the TOTAM study. Of those patients, 11 patients were excluded from this analysis due to (i) non-adherence (*n* = 1) and (ii) non-steady-state endoxifen concentration (*n* = 10). Geno- and phenotyping was successfully performed in almost all of the patients and conform Hardy–Weinberg equilibrium (*p* < 0.05). Hot flashes (61%), arthralgia (19%), fatigue (11%), vaginal dryness (8%), and mood swings (6%) were the most commonly reported tamoxifen-related adverse events (all CTCAE grade 1) during the first six months after initiation of tamoxifen treatment. Patients’ characteristics are shown in Table 1. No statistically significant differences were found between the full cohort (*n* = 303) and patients with complete information (*n* = 281) concerning patient characteristics. Tamoxifen and endoxifen plasma concentrations were measured from patients prescribed 20 mg once daily and who reached steady-state concentration. Median (IQR) steady-state trough plasma concentration of tamoxifen and endoxifen were 308 (248.0–385.5) nmol/L and 26.2 (17.0–35.3) nmol/L, respectively.

**Table 1:** Patient characteristics at baseline, *n *= 303

Characteristic	*N* (%) or Median (IQR)	
Age (years)	56	(47–65)
Weight (kg)	73.5	(65.3–84.0)
BMI (kg m^−2^)	26	(22.8–29.7)
Tamoxifen (nmol/L)	308	(248.0–385.5)
Endoxifen (nmol/L)	26.2	(17.0–35.3)
CYP2D6, phenotype		
PM	25	(8.2)
IM	99	(32.7)
NM	177	(58.4)
Missing data	2	(0.7)
CYP2D6 inhibitor		
Weak inhibitor	4	(1.3)
Strong inhibitor	1	(0.3)
No inhibitor	298	(98.4)
CYP3A4*22 genotype		
CC	269	(88.8)
CT/TT	28	(9.2)
Missing data	6	(2)
CYP3A4/5 inhibitors		
Weak inhibitor	12	(4)
No inhibitor	291	(96)
Morisky Medication Adherence Scale		
High adherence (n/N)	132	(91)
Medium adherence (n/N)	7	(5)
Low adherence (n/N)	6	(4)
Missing data	158	(52.1)

*BMI* Body Mass Index, CYP2D6 strong inhibitor: quinidine; CYP2D6 weak inhibitors: escitalopram, sertraline, and citalopram; CYP3A weak inhibitors: pantoprazole, prednisone and omeprazole. *PM* poor metabolizer; *IM* intermediate metabolizer; *NM* normal metabolizer

### Impact of genetic polymorphisms on blood plasma concentrations

**Fig. 2:**
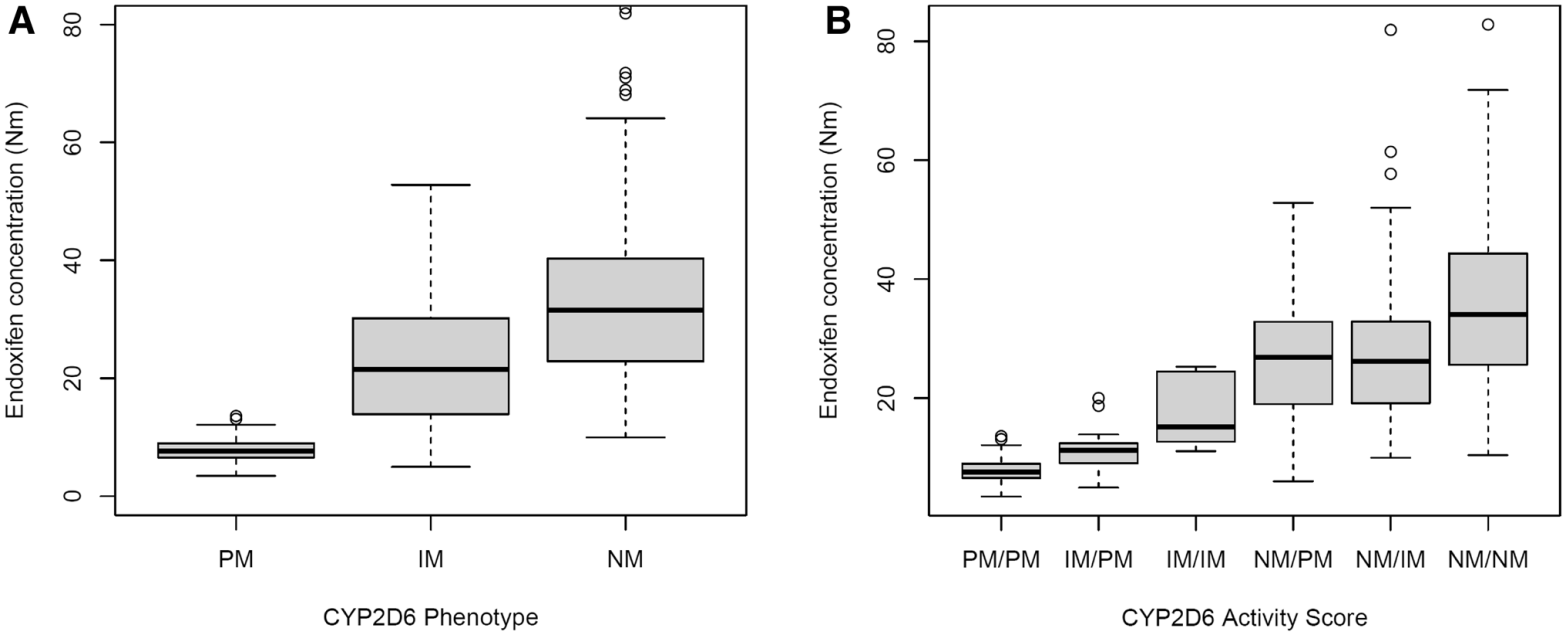
**A** Pharmacokinetic profile of endoxifen steady-state concentrations (*n* = 301) stratified based on CYP2D6 phenotype, median (IQR). *PM* poor metabolizer 7.6 (6.6–9.0); IM, intermediate metabolizer 21.5 (13.9–30.2); NM, normal metabolizer 31.5 (22.9–40.3). **B** Pharmacokinetic profile of endoxifen steady-state concentrations (*n* = 301) stratified on CYP2D6 activity score (AS) based on their allele combination, median (IQR). PM/PM 7.6 (6.6–9.0), IM/PM 11.2 (9.1–12.4), IM/IM 15.2 (13.3–24.1), NM/PM 26.6 (19.0–32.8), NM/IM 26.6 (19.2–32.8), NM/NM 34.1 (25.6–44.3)

Inter-individual variability in plasma concentration was observed for tamoxifen and endoxifen, ranging from 127 to 881 nmol/L and 3.4–82.8 nmol/L, respectively. The median endoxifen concentration in patients with an NM 31.5 (22.9–40.3) nmol/L was statistically significantly higher than in patients with an IM 21.5 (13.9–30.2) nmol/L and PM phenotype 7.6 (6.6–9.0) nmol/L; *p* < 0.001, respectively. Results of different pharmacokinetic profiles are depicted in both Fig. 2A (stratified on CYP2D6 phenotype) and Fig. 2B (stratified on CYP2D6 activity score) Consecutively, based on CYP2D6 phenotype 100% of PMs, 32% of IMs, and 7% of NMs had endoxifen concentration (< 16 nmol/L, a threshold often used in the literature).

CYP3A4*22 genotyping showed no statistically significant difference in median endoxifen concentrations between CYP3A4*22 carriers CT/TT and wildtype CC (*p* = 0.31). Nevertheless, a statistically significant difference was found in median tamoxifen concentrations in CYP3A4*22 carriers 386 (296.0–455.8) nmol/L compared to wildtype 303 (242.0–378.0) nmol/L)—indicating carriers had a higher tamoxifen concentration (*p* < 0.001).

### Predictors of blood plasma concentrations

Multiple linear regression was performed to determine statistically significant predictors of endoxifen. To adjust for potential bias due to missing data, MICE was considered and presented as primary analysis. Approximately seven percent of all patients had missing information. The missingness mechanism was assumed to be missing at random (MAR). Given the limited amount of missing data, the number of imputed datasets was chosen to be *m* = 10 [32, 38]. Adherence to tamoxifen treatment was left out in the primary analysis due to low representation of the study population; adherence was only questioned in the first 145 participants of the trial. However, adherence to tamoxifen treatment was found high in 91% of all participants, and 5% and 4% of those showed medium and low adherence, respectively.

The variable selection procedure across all intermediate MI datasets identified age, weight, BMI, and *CYP2D6* as statistically significant predictors. Comedication (CYP2D6/ CYP3A4 inducers or inhibitors), intake with food and CYP3A4*22 genotype were excluded, see Supplementary material (Sect. 5.1) for more details of the selection procedure. Wald statistics were computed to assess significance of variables in our variable selection procedure. Therefore, two nested models including and excluding BMI and intake-time-of-the-day were compared, leading to BMI and intake-time-of-the-day being excluded from the final model, as the Wald-based test statistics were non-significant (*p* = 0.14; *p* = 0.56). Pooled parameter estimates from the imputed datasets and accompanying average bootstrap-based 95% CIs of the model parameter estimates and average-based optimism-adjusted *R*^2^ values were summarized in Table 2.

**Table 2:** Primary analysis, multiple linear regression estimates, and average-based optimism-adjusted *R*^2^

Coefficient	Estimate	Std. error	Pr ( >|t|)	95% CI lower bound	95% CI upper bound	Bootstrap CI lower bound	Bootstrap CI Upper bound
Intercept	2.178	0.172	< 0.001***	1.843	2.500	1.870	2.497
CYP2D6							
IM/PM	0.275	0.178	< 0.001***	0.034	0.492	0.034	0.487
IM/IM	0.734	0.152	< 0.001***	0.424	1.029	0.404	1.033
NM/PM	1.060	0.149	< 0.001***	0.867	1.221	0.910	1.202
NM/IM	1.116	0.152	< 0.001***	0.937	1.283	0.965	1.263
NM/NM	1.375	0.170	< 0.001***	1.208	1.536	1.220	1.506
Age	0.006	0.002	< 0.001***	0.003	0.010	0.003	0.012
Weight	− 0.005	0.002	0.002**	− 0.007	− 0.002	− 0.008	− 0.002
Adjusted *R*^2^	0.571					0.491	0.635

Pr ( >|t|), probability of observing any value equal or larger than t; CI, confidence interval; *, p-value < 0.05; **, p-value < 0.01; ***, p-value < 0.001; NM, normal metabolizer; IM, intermediate metabolizer; PM, poor metabolizer

Thus, the final model included age, weight, and *CYP2D6* genotype to be statistically significant predictors of endoxifen concentration. Altogether, these predictors explained 57% (95% CI 0.49–0.64) of the total inter-individual variability. *CYP2D6* genotype accumulated for approximately 54%, whereas in addition age and weight only explained 1.8% and 1.5% of the inter-individual variability, after adjusting for *CYP2D6* genotype status, respectively. Complete case analysis identified, similarly, age, weight, and *CYP2D6* genotype as statistically significant predictors. However, no meaningful difference was found in parameter estimates and optimism-adjusted *R*^2^ values between primary analysis and CCA (Table 3). Details with regard to model selection and diagnostics are presented in the supplementary material (Sect. 5.1 and 5.2).

**Table 3:** Sensitivity analysis and complete case analysis

Coefficient	Estimate	Std. error	Pr ( >|t|)	95% CI lower bound	95% CI upper bound	Bootstrap CI Lower bound	Bootstrap CI Upper bound
Intercept	2.178	0.172	< 0.001***	1.839	2.517	1.884	2.465
CYP2D6							
IM/PM	0.275	0.124	< 0.027*	0.031	0.519	0.045	0.459
IM/IM	0.734	0.162	< 0.001***	0.414	1.053	0.435	0.982
NM/PM	1.060	0.090	< 0.001***	0.890	1.234	0.888	1.183
NM/IM	1.116	0.090	< 0.001***	0.942	1.291	0.960	1.251
NM/NM	1.374	0.084	< 0.001***	1.207	1.542	1.232	1.505
Age	0.006	0.001	0.001**	0.002	0.010	0.003	0.010
Weight	− 0.005	0.001	0.002**	− 0.008	− 0.001	− 0.008	− 0.002
Adjusted *R*^2^	0.556					0.469	0.641

Pr ( >|*t*|), probability of observing any value equal or larger than *t*

*CI* confidence interval;* NM* normal metabolizer; *IM* intermediate metabolizer; *PM* poor metabolizer

*p-value < 0.05; **p-value < 0.01; ***p-value < 0.001

### Predictors of subtherapeutic blood plasma concentration

**Fig. 3:**
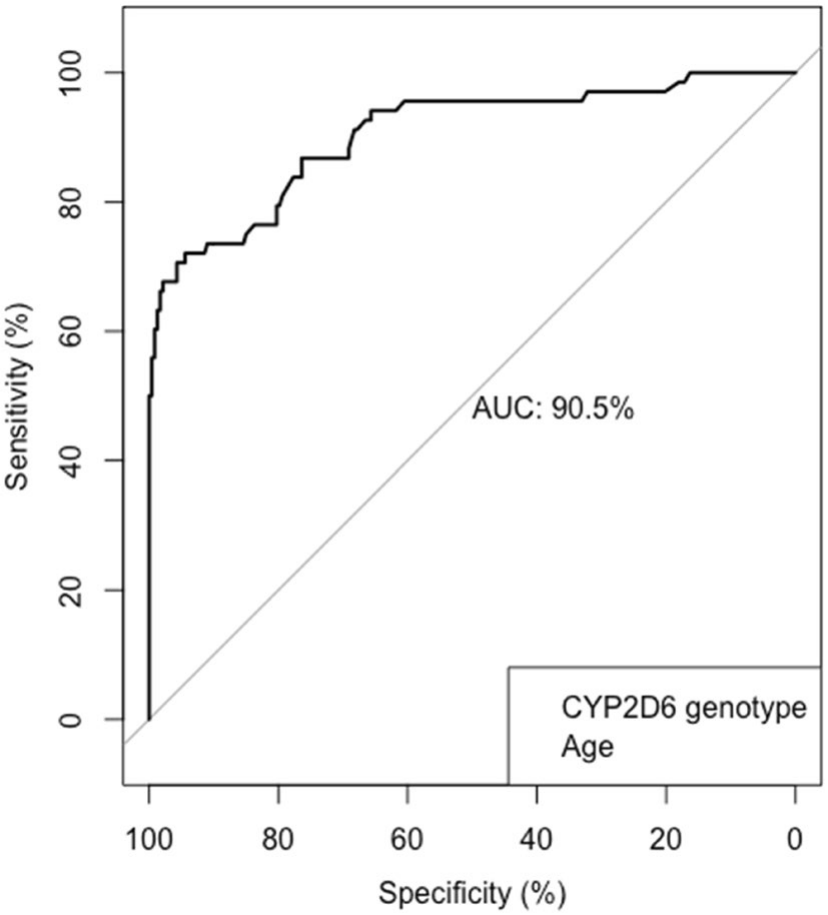
Receiver operator curve and area under the curve

The extent of missing information in key predictors found by multiple linear regression was approximately 0.6%. Therefore, an imputation model was not considered, and analysis was performed accordingly. Key predictors for subtherapeutic risk of endoxifen (< 16 nmol/L) were age and *CYP2D6* genotype. A bootstrap-based area under the curve (AUC) was computed and showed an optimism-adjusted AUC of 90% (95% CI 0.86–0.95; Fig. 3). Considering an optimal cut-off probability of 0.8, sensitivity, and specificity of the model were estimated to be 66% and 98%, respectively. A goodness-of-fit test indicated a good model fit given the Hosmer–Lemeshow test statistic was non-significant (*p* = 0.28). Parameter estimates by logistic multiple regression model and accompanying bootstrap-based 95% CIs were depicted in Table 4.

**Table 4:** Logistic regression for subtherapeutic endoxifen levels (< 16 nmol/L)

Coefficient	Estimate	Standard error	Pr ( >|t|)	95% CI Lower bound	95% CI Upper bound	Bootstrap CI lower bound	Bootstrap CI upper bound
Intercept	− 2.088	1.050	0.04*	− 4.267	− 0.110	− 17.029	− 0.146
CYP2D6							
NM/IM	1.717	0.690	0.01*	0.460	3.256	0.143	3.396
NM/PM	1.793	0.680	0.008**	0.559	3.321	0.153	3.500
IM/IM	3.950	0.946	< 0.001***	2.182	5.978	− 0.099	20.908
IM/PM	5.780	0.965	< 0.001***	4.108	7.990	3.825	22.310
PM/PM	22.223	1293.752	0.986	− 24.296	496.5	20.370	24.420
Age	− 0.028	0.017	0.10	− 0.062	− 0.005	− 0.056	0.001

Pr ( >|*t*|), probability of observing any value equal or larger than *t*

*CI* confidence interval;* NM* normal metabolizer; *IM* intermediate metabolizer; *PM* poor metabolizer

*p-value < 0.05; **p-value < 0.01; ***p-value < 0.001

## Discussion

Our data showed a high inter-individual variability in both tamoxifen and endoxifen concentrations. Age, weight, and *CYP2D6* genotype were identified as statistically significant predictors of endoxifen plasma concentrations. Consequently, a large proportion of the variability remains unexplained by the model at hand, suggesting the presence of additional unobserved predictors affecting true patient-specific endoxifen concentrations. By means of logistic regression, ROC analyses showed an optimism-adjusted AUC of 90% (95% CI 0.86–0.95), thereby indicating an excellent predictive accuracy of subtherapeutic endoxifen concentration (< 16 nmol/L) with *CYP2D6* genotype and age as statistically significant predictors. However, the model showed a sensitivity and specificity of 66 and 98 percent, respectively, indicating a high probability of (misclassification) error for the patients with subtherapeutic endoxifen concentrations. Consecutively, those patients will have a high false negative rate and thereby potentially misclassified.

The results of this study confirm that *CYP2D6* genotype accounts for a large proportion of the variability and has high predictive properties for identifying subtherapeutic endoxifen concentrations. Comparable results were found in estimates and R^2^ values by colleagues Teft et al*.* and Schroth et al*.* explaining 39 to 58 percent of the inter-individual variability in endoxifen concentrations [15, 39]. Additionally, other iso-enzymes of the cytochrome P450 system significantly affects endoxifen formation [16, 19, 26]. Puszkiel et al*.* investigated the effects of CYP3A4*22, CYP2C19*2, and CYP2B6*6 on endoxifen concentrations. CYP3A4*22 homozygous and heterozygous patients were associated with a 16–25% higher endoxifen concentration compared to wild type, irrespective of *CYP2D6* genotype. However, no significant differences were found. CYP2C19*2 and CYP2B6*6 showed only marginal effects on endoxifen concentrations, indicating minimal clinical value [40]. Thus, *CYP2D6* is evidently an important factor in predicting endoxifen concentrations.

Age and weight were identified as statistically significant predictors, but only explained an small (1.8 and 1.5) percent of the total variability with small effect sizes after adjusting for CYP2D6 genotype, respectively. Our data implied a positive association between age and endoxifen—which corresponds with other published model-based analyses [25, 41]—yet only explained an additional 1.8 percent of the total inter-individual variability including small effect sizes (0.006; 95% CI 0.003–0.010). Puszkiel and colleagues reported a negative association between age and endoxifen leading to a lower endoxifen exposure if age increased. [37] However, taking into account their substantial sample size (> 900), only approximately 5% of the population was above 65 years old and therefore limits the interpretation in other populations. Consequently, conflicting results regarding the impact of age with either no association, increased endoxifen concentrations or decreased endoxifen concentrations in older patients have been reported [39–41].

Weight has been described as a significant predictor for the formation of endoxifen. In the analysis by Mueller-Schoell et al*.*, body weight was found to be negatively correlated with endoxifen concentrations [25]. Additionally, body weight was also in other analyses identified as relevant covariate and a risk factor for subtherapeutic concentrations [9, 40]. Our data also implied a negative association in the multiple linear regression analysis between body weight and endoxifen concentration and only explained an additional ≈ 1.5% of the total variability including small effect sizes (− 0.005; 95% CI − 0.008 to − 0.002). Interestingly, Mueller-Schoell et al*.* identified participants with high body weight at increased risk of subtherapeutic endoxifen. Up to 13-fold differences in endoxifen concentration were found in heavy young (22 years, 150 kg) and light elderly (95 years, 39 kg). However, analysis was done across extreme values and the risk shrunk after averaging across the population [25]. Additionally, and similarly, after multivariate analysis CYP2D6 genotype, age, and weight were identified as significant predictors. However, the unexplained variability in endoxifen remains high and therefore predictions—as made by various models—may deviate in real life [25, 40, 41].

A strength of this study lies in the statistical analysis. MI was used to minimalize bias by missing data (under the assumption of MAR) and results of both primary analysis and CCA were presented. Additionally, by incorporating MI, the statistical power increased by using the full extent of the dataset. This approach favors CCA as primarily used in the methodologies in other relevant prediction studies. Mixed-effect association methods—used by Mueller-Schoell et al., and Klopp-Schulze et al., to predict endoxifen concentration—may prevent false positive associations and increase power in comparison to multiple regression analysis [25, 41]. Nonetheless, in our model selection procedures across multiple imputed datasets the probability of type-I error is decreased by the application of validated techniques (SM: Sect. 5.1 and 5.2). Moreover, our prediction model performance was quantified relying on a bootstrap approach to reduce model optimism and to function as internal validation of the prediction model thereby providing a more realistic estimate of its performance estimates. The results of the primary analysis and the CCA showed marginal differences. These marginal differences are likely caused by the low proportion of missing data (≈ 7%) in the total dataset and even an even lower proportion of missing data (≈ 1%) amongst the predictors included in the model.

Earlier research showed, fairly extensive use of important inhibitors and inducers in the general ER+ breast cancer population. Primarily, concurrent treatment with CYP2D6 inducers may significant decrease endoxifen concentration and should be monitored, e.g., selective serotonin reuptake inhibitors (SSRI) are known to decrease endoxifen concentration [15, 16, 19]. Therefore, concurrent treatment with, especially, CYP2D6 inhibitors should be monitored and possibly intervened accordingly—to sustain tamoxifen efficacy.

Additionally, 91% of our population showed high adherence to tamoxifen treatment whereas five and four percent of the participants showed medium and low adherence, respectively. Likely, this was an advantage for model building as under these conditions a low proportion of the variability is likely to be attributed by the degree of adherence. However, these results lack external validity as adherence is a major issue in the adjuvant treatment with tamoxifen. Pistilli et al*.* [18] showed that after one-year the adherence to tamoxifen is 86% based on serum concentration and self-declared adherence (95% CI 84–88%). Nevertheless, real-life adherence estimations range from 15 to 72% non-adherence over 5 years of tamoxifen therapy [20, 21].

Alternatively, the therapeutic drug monitoring (TDM) strategy might be of benefit for this population as pragmatic approach [42]. Personalizing tamoxifen treatment by dose adjustments based on measured steady-state concentration may benefit the population at risk for subtherapeutic endoxifen. Currently, endoxifen concentration is not a validated biomarker for tamoxifen efficacy. Ideally, to demonstrate the clinical benefit of TDM it is desirable to perform a randomized controlled trial. However, the feasibility of such a study is very low given the extreme high number of patients required and the long follow-up period and required endpoints i.e., death and recurrence [43]. On the other hand, positive results of a tamoxifen feasibility study and cost-effectiveness evaluations are encouraging for multidisciplinary discussions about implementation in clinical practice [44]. Klopp-Schulze et al. identified *CYP2D6*, drug-drug interactions and age as significant predictors of endoxifen. However, and similarly, the unexplained inter-individual variability in endoxifen concentration remained large (47.2%) and therefore they concluded that therapeutic drug monitoring may be a beneficial strategy.

A combination of CYP2D6 predicted phenotype-guided dosing and therapeutic drug monitoring at steady-state concentration was proposed, including Bayesian forecasting to test different doses (off label; > 40 mg tamoxifen once daily) [41]. Our published primary TOTAM data indicating that PMs might benefit with a maximal daily dose of 40 mg as described in the tamoxifen drug label combined with TDM; and IMs might benefit with the standard dose combined with TDM. For NMs 20 mg, once daily tamoxifen might be sufficient without TDM for most of the tamoxifen users [45]. As a result of earlier research, almost all concomitant moderate and strong CYP2D6 inhibitors are included in the medication monitoring system of Dutch pharmacies as a monitoring signal [46]. Hence, minimal use of concomitant CYP2D6 inhibitors was noticed in our population thereby no association between endoxifen concentration and comedication was found likely due lack of statistical power.

In conclusion, the inter-individual variability of endoxifen concentration is largely explained by CYP2D6 genotype and for a small proportion by age and weight. However, small effect sizes accompanied with a high remaining unexplained inter-individual variability were found. Furthermore, our prediction model showed a sensitivity and specificity of 66 and 98%, respectively, thereby indicating that other yet unknown parameters influence endoxifen plasma steady-state concentrations. In other words, this analysis shows that only model-guided tamoxifen dosing is not sufficient in clinical practice for tamoxifen precision dosing. Therefore, we recommend model-guided tamoxifen dosing in combination with therapeutic drug monitoring—by directly measuring endoxifen concentration—as a practical tool to personalize tamoxifen treatment.

## Supplementary Information

Below is the link to the electronic supplementary material.

Supplementary file1 (DOCX 43 kb)
