# Supplementary material for: Factors affecting inter-individual variability in endoxifen concentrations in patients with breast cancer: results from the prospective TOTAM trial
Source: Breast Cancer Res Treat. 2022 Jul 16;195(1):65–74. doi: 10.1007/s10549-022-06643-y (PMC9338137; doi:10.1007/s10549-022-06643-y)
Supplement: Supplementary file 1 — Supplementary file1 (DOCX 43 kb) [file 10549_2022_6643_MOESM1_ESM.docx]

# **Supplementary Material**

- 1. **Model selection procedure across multiple imputed datasets**

Model selection was incorporated in the *m* multiple imputed (MI) and complete datasets by the MICE algorithm. Model selection was done on each MI dataset separately by means of backwards selection for multiple linear regression and based on two stopping rules – namely a significance level of p<0.10 and AIC (32–34). Additionally, a majority rule is applied. More specifically, predictors were included in the final model when they were selected in at least 50% of the intermediate models across all MI datasets. Thereby, the majority rule is used as a variable importance measure by excluding variables that have a low selection probability and including those variables with a high selection probability. Accordingly, variables with a probability of <0.4 were considered as having a low selection probability, variables with a probability of ≥0.4 and <0.6 were considered as having an intermediate selection probability, and variables with a probability of ≥0.6 were considered as variables with a high selection probability. Inherently, this method prevents unimportant variables, with a selection probability of <0.4, to be included in the model and operates as a correction for multiple testing and inflation of the (family-wise) Type I error (32).

Although the majority rule provides insight in the variability of models between the imputed datasets, it is a method that does not incorporate the uncertainty caused by missing observations that have been remediated in each of the MI datasets. Therefore, Wald-based statistics were computed for variables with selection probabilities ranging between 0.4 and 0.6 in order to compare two nested models for improvement. This approach therefore leads to the exclusion of non-predictive variables (32,35). Variables with a selection probability of ≥0.6 were directly included. Analyses across intermediate MI datasets showed that age, BMI, *CYP2D6*, CYP2D6 inhibitors, intake-time-of-the-day, and weight were relevant predictors across ten, five, ten, one, four, and ten out of the total ten MI datasets, respectively. Therefore, age, *CYP2D6* and weight were directly included and CYP2D6 inhibitor use was excluded. Two nested models including and not including BMI and intake-time-of-the-day were compared leading to BMI and intake-time-of-the-day being excluded from the model given the Wald-based test statistic was non-significant (p=0.14; p=0.56). Conclusively, the final model incorporated age, *CYP2D6* and weight as statistically significant predictors for endoxifen concentration.

- 1. **Model diagnostics**

Goodness-of-fit plots were made to assess model performance and to check model assumptions such as normality, linearity, no or minimal collinearity, and heteroscedasticity of residuals. A Shapiro-Wilk test showed a significant deviation from normality for endoxifen concentration, after correction for covariate information (p<0.001). However, in large datasets such a test is highly sensitive for small deviations from normality and therefore likely to lead to a significant result. Therefore, Q-Q plots were analyzed for all intermediate models across MI datasets which showed no visual deviation from normality in endoxifen concentration. Additionally, residuals vs. fitted values were plotted and a Scale-Location plot was computed which both showed linearity and no indication of heteroscedasticity, i.e., unequal along the regression line. No multicollinearity was found across variables in the final models by means of variance inflation factors, both for logistic and linear regression analyses.

Often it is more advantageous to assess the distributional discrepancy between the observed and imputed data (32,47). A good fit should represent a similar distribution between observed and imputed data i.e., the imputation could be considered as real values. An exception can be made when the missing mechanism is considered to be MCAR. Consecutively, summary statistics including mean, median and spreads could be computed to assess differences. Additionally, distributional differences between observed and imputed data could be assessed. However, due to the low proportion of missing data, such analysis is not feasible. Key predictors of endoxifen; age, weight and *CYP2D6* genotype consisted of approximately 0.6 percent missing information across all participants. Therefore, the imputations will not be able to create any deviations in the distribution.
